# Comparative evaluation of disease dynamics in wild boar and domestic pigs experimentally inoculated intranasally with the European highly virulent African swine fever virus genotype II strain “Armenia 2007”

**DOI:** 10.1186/s13567-024-01343-5

**Published:** 2024-07-15

**Authors:** Pedro J. Sánchez-Cordón, Fabian Z. X. Lean, Carrie Batten, Falko Steinbach, Aleksija Neimanis, Marie-Frédérique Le Potier, Emil Wikström-Lassa, Felicity Wynne, Rebecca Strong, Stephen McCleary, Helen Crooke, Dolores Gavier-Widén, Alejandro Núñez

**Affiliations:** 1 Department of Pathology and Animal Sciences, Animal and Plant Health Agency (APHA-Weybridge), New Haw, Addlestone, UK; 2 The Pirbright Institute, Pirbright, Woking, UK; 3 Department of Virology, Animal and Plant Health Agency, (APHA-Weybridge), New Haw, Addlestone, UK; 4 Department of Comparative Biomedical Sciences, School of Veterinary Medicine, Faculty of Health and Medical Sciences, University of Surrey, Guildford, UK; 5 Department of Pathology and Wildlife Diseases, Swedish Veterinary Agency (SVA), Uppsala, Sweden; 6 ANSES, Laboratoire de Ploufragan/Plouzané/Niort, Unité Virologie Immunologie Porcines, Anses, 22440 Ploufragan, France; 7 Present Address: Department of Infectious Diseases and Global Health, Centro de Investigación en Sanidad Animal, Instituto Nacional de Investigación y Tecnología Agraria y Alimentaria, Consejo Superior de Investigaciones Científicas, Valdeolmos, Madrid, Spain; 8 Present Address: Department of Pathobiology & Population Sciences, The Royal Veterinary College, Hawkshead Lane, North Mymms, Hatfield, AL9 7TA UK

**Keywords:** African swine fever virus (ASFV), domestic pigs, wild boar, clinical course, disease outcome, macroscopic lesion evolution, virus dynamics

## Abstract

Since the reintroduction of African swine fever virus (ASFV) in Europe in 2007 and its subsequent spread to Asia, wild boar has played a crucial role in maintaining and disseminating the virus. There are significant gaps in the knowledge regarding infection dynamics and disease pathogenesis in domestic pigs and wild boar, particularly at the early infection stage. We aimed to compare domestic pigs and wild boar infected intranasally to mimic natural infection with one of the original highly virulent genotype II ASFV isolates (Armenia 2007). The study involved euthanising three domestic pigs and three wild boar on days 1, 2, 3, and 5 post-infection, while four domestic pigs and four wild boar were monitored until they reached a humane endpoint. The parameters assessed included clinical signs, macroscopic lesions, viremia levels, tissue viral load, and virus shedding in nasal and rectal swabs from day 1 post-infection. Compared with domestic pigs, wild boar were more susceptible to ASFV, with a shorter incubation period and earlier onset of clinical signs. While wild boar reached a humane endpoint earlier than domestic pigs did, the macroscopic lesions were comparatively less severe. In addition, wild boar had earlier viremia, and the virus was also detected earlier in tissues. The medial retropharyngeal lymph nodes were identified as key portals for ASFV infection in both subspecies. No viral genome was detected in nasal or rectal swabs until shortly before reaching the humane endpoint in both domestic pigs and wild boar, suggesting limited virus shedding in acute infections.

**Supplementary Information:**

The online version contains supplementary material available at 10.1186/s13567-024-01343-5.

## Introduction

African swine fever (ASF) has remained one of the most important threats to the global pig industry in recent decades, with more than 50 countries across Europe, Asia, the Caribbean and Africa being affected [1]. The causative agent, African swine fever virus (ASFV), is a large and complex double-stranded DNA arbovirus, the sole member of the *Asfarviridae* family within the genus *Asfivirus* [2]. Currently, there are 24 distinct genotypes [3]. The disease affects both domestic and wild suids of various breeds and ages, resulting in substantial lethality rates exceeding 90% among susceptible animals. Notably, soft ticks from the *Ornithodoros* genus, including *O. moubata* and *O. erraticus*, have been highlighted as reservoirs and carriers of ASFV [4]. Additionally, transmission routes, including direct contact, contact with wild boar and aerosols, are important, particularly in the context of the disease situation, which has continued since 2007 [5]. Efforts to develop effective ASF vaccines face significant challenges, exemplified by a recent live attenuated vaccine surprisingly approved for commercialisation and use in Vietnam [6], albeit with many doubts about its efficacy and safety in the field.

Significant progress has been made in controlling ASF within domestic pig populations, primarily through robust biosecurity measures. However, the challenge posed by wild boar as notable reservoirs in Europe and Asia persists, as they threaten the food chain, hinder eradication efforts and contribute to the geographical spread of the disease. In vivo studies have shown that wild boar are more susceptible to ASFV infection than are domestic pigs [7–9]. For instance, oral and intramuscular experimental infections of adult wild boar with genotype II isolates from the Caucasus region (Armenia in 2008 and Chechen Republic in 2009) resulted in acute disease, with 100% lethality in less than 10 days, without a serological response [7, 10]. While limited shedding of ASFV through nasal discharge or faeces has been reported, efficient transmission to in-contact wild boar is evident, leading to acute disease in the recipients. Furthermore, differences in disease outcomes in wild boar infected with lower-virulence ASFV variants in Europe [11] also increase the complexity of our understanding of ASFV pathobiology in wild boar.

Since the incursion of genotype II in the Caucasus region in 2007, most studies have focused primarily on assessing the impact of ASFV strains belonging to genotype II in domestic pigs [8, 9, 12–15]. More contemporaneous genotype II strains, such as those from Estonia 2014 or Belgium 2018/1, have been used in the experimental infection of both domestic pigs and wild boar [9, 16–19]. In contrast, the original isolates obtained in the Caucasus region after the reintroduction of the disease in Europe have been investigated in only a few in vivo studies in wild boar [7, 8, 10, 20]. The diversity of genotype II ASFV strains has evolved over time due to viral mutations and recombination. However, the lack of experimental data on the ancestral isolates of genotype II ASFV limits our understanding of the changes in infection dynamics. Furthermore, extensive research on the pathogenic mechanisms of the disease has only been performed using high- and moderate-virulence ASFV isolates of genotype I, which emerged after the introduction of the ASFV to the Iberian Peninsula during the 1950s and [21]. In contrast, the understanding of disease pathogenesis and the resulting immune response against genotype II strains remains limited in domestic pigs and is even less explored in wild boar. Currently, there is only one published comparative study that examines the infection kinetics in wild boar and domestic pigs following oronasal infection with a moderately virulent genotype II isolate [17].

In both domestic pigs (*Sus scrofa domesticus*) and wild boar (*Sus scrofa spp*.), in vivo experimental results have shown variations in the outcomes and severity of clinical disease following infections with the prevailing ASFV genotype II strains from Europe. These variations, often exceeding expected biological differences, may be attributed to differences in experimental conditions. For instance, the intramuscular route has been widely employed for experimental in vivo ASFV research due to its ability to reproduce clinical disease. However, it bypasses the mucosal surfaces and innate defence mechanisms encountered during natural infection. Experimental oronasal infections more accurately represent natural infection and early clinical disease dynamics and pathogenic mechanisms [22].

The objective of this study was to evaluate and compare the pathogenesis of ASFV in domestic pigs and wild boar starting at 24 h post-intranasal infection using Armenia 2007, one of the original highly virulent genotype II isolates that emerged after disease reintroduction in the Caucasus region. This study sought to understand the initial disease dynamics in both subspecies. Specifically, we compared various aspects, including disease progression, macroscopic lesions, viremia levels, virus shedding, and virus load within target organs. This knowledge provides valuable information to aid in early diagnosis and to contribute to the evaluation and development of new vaccines essential for effectively preventing and combating the spread of ASF.

## Materials and methods

### Virus

The highly virulent ASFV genotype II isolate used (Armenia 2007) was kindly provided by the EU reference laboratory for ASF (CISA-INIA/CSIC, Valdeolmos, Madrid Spain). Stocks for inoculation were grown in primary porcine blood peripheral monocytes, and viral titres were determined as the amount of virus causing haemadsorption in 50% of inoculated cultures (HAD_50_/mL).

### In vivo experimental design

In vivo experiments were carried out in containment facilities at the Animal and Plant Health Agency (APHA, Weybridge, UK). These experiments were reviewed by the APHA Animal Welfare and Ethical Review Board and conducted in accordance with the UK Animals (Scientific Procedures) Act 1986 under project licence PF971B5E3.

**Figure 1:**
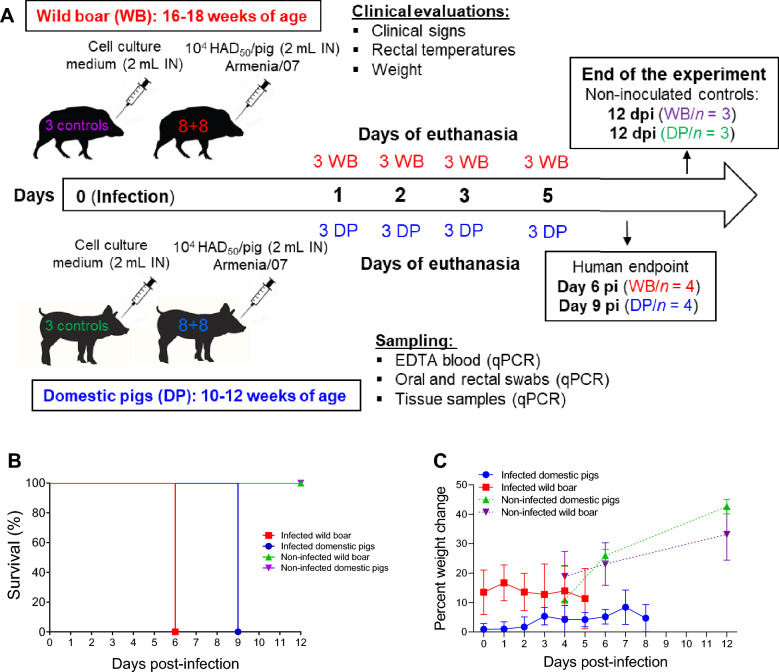
**Experimental design, survival rate and weight assessment. A** Infection and euthanasia schedule. Nineteen commercial domestic Large White/Landrace cross pigs (DPs) of both sexes, aged 10–12 weeks at the commencement of the experiment, were used alongside nineteen farmed European wild boar (WBs) of both sexes, aged 16–18 weeks. Animals were randomly allocated into four groups consisting of 8 animals (either wild boar or domestic pigs) that were intranasally inoculated (IN) with a dose of 10^4^ HAD_50_/pig of the highly virulent ASFV genotype II isolate ASFV isolate “Armenia 2007” (1 mL per nostril). Two additional groups consisting of 3 domestic pigs or 3 wild boar were used as mock-inoculated controls and received the same volume of cell culture medium via the intranasal route. On days 1, 2, 3, and 5 post-infection (pi), six animals that had been randomly assigned beforehand (comprising 3 domestic pigs and 3 wild boar) were euthanised. The remaining inoculated animals (4 pigs and 4 wild boar) were euthanised and necropsied upon reaching predetermined humane endpoints on day 6 (all remaining wild boar) and day 9 pi (all remaining domestic pigs). Mock-inoculated control animals were euthanised at the conclusion of the experiment (day 12 pi). **B** Mortality of infected wild boar (*n* = 4) and infected domestic pigs (*n* = 4) that were kept alive until a humane endpoint was reached. Mock-infected animals are also shown. **C** Comparative weight assessment between infected and noninfected animals.

Nineteen commercial domestic Large White/Landrace cross pigs of both sexes, aged 10–12 weeks at the commencement of the experiment, were used alongside nineteen farmed European wild boar of both sexes, aged 16–18 weeks, and sourced from a commercial provider (Figure 1A). Following a 7-day acclimatisation period, a total of thirty-eight animals (19 domestic pigs and 19 wild boar) were randomly allocated into four groups consisting of 8 animals each (either wild boar or domestic pigs), which were assigned to the infected groups. Two additional groups consisting of either 3 domestic pigs or 3 wild boar were assigned as mock-inoculated controls. Animals were assigned to groups with similar average weights and similar distributions of males and females. To prevent potential aggression, domestic pigs and wild boar were housed separately. As described in Figure 1A, the animals were euthanised at predetermined time points following infection prior to inoculation.

To mimic natural infection, all animals were sedated with Stresnil and then intranasally inoculated with a dose of 10^4^ HAD_50_/pig of the virulent ASFV isolate “Armenia 2007”. One millilitre of inoculum was administered per nostril using intranasal mucosal atomisation devices (MAD Intranasal, Teleflex). To ensure thorough administration of the inoculum, the animals’ heads were maintained in an upright position for 1 min post-inoculation. On the day of inoculation, the inoculum was titrated to verify the administered dose. The mock-inoculated controls were housed separately and subjected to mock infection using identical procedures, but RPMI cell culture medium (Gibco) was used in place of the viral inoculum.

The day of experimental infection was designated day 0. All animals were weighed before the experimental inoculation (day 0). Throughout the experiment, rectal temperatures were measured once daily, and clinical signs were monitored daily using a previously described scoring system [23]. The sum of the points was recorded as the clinical score, which was also used to establish humane endpoints before commencing the experiment. On days 1, 2, 3, and 5 post-infection (pi), six animals that had been randomly assigned beforehand (comprising 3 domestic pigs and 3 wild boar) were sedated and euthanised by the administration of barbiturate (Figure 1A). The remaining inoculated animals (4 pigs and 4 wild boar) were euthanised and necropsied upon reaching predetermined humane endpoints on day 6 (all remaining wild boar) and day 9 pi (all remaining domestic pigs). Mock-inoculated control animals (*n* = 6) were euthanised at the conclusion of the experiment (day 12 pi).

### Sampling and macroscopic evaluation of lesions

Blood samples were collected into vacutainers containing EDTA (BD biosciences) from the anterior vena cava of all animals (inoculated and non-inoculated) before inoculation (day 0). Following inoculation, blood samples, along with nasal and rectal swabs, were obtained at the scheduled euthanasia time points on days 1, 2, 3, and 5 pi, as well as at the humane endpoint (day 6 pi for wild boar and day 9 pi for domestic pigs). Additional blood samples and swabs were also taken from the remaining infected domestic pigs on day 8 pi. For the mock-inoculated control animals, blood samples and swabs were collected on day 8 pi and at the end of the experiment on day 12 pi. Blood samples and swabs were frozen at −80 °C for subsequent detection of the ASFV genome by quantitative PCR (qPCR).

All euthanised animals underwent necropsies, during which macroscopic evaluations were conducted in accordance with a previously established macroscopic scoring protocol [24]. Macroscopic lesions were scored and individually documented, representing the cumulative score observed across various organs in the examined animals.

Additionally, tissue samples were collected from the palatine tonsils, lungs (right cranial lobe), spleen, medial retropharyngeal, tracheobronchial, and gastrohepatic lymph nodes, and these samples were frozen at −80 °C for subsequent detection of the ASFV genome through qPCR.

### Quantification of viral DNA levels in blood, tissues and nasal swabs

DNA was extracted in duplicate from 100 µL of EDTA blood, tissue homogenate, or swab eluate using the MagVET extraction kit on the KingFisher Flex automated extraction system (ThermoFisher, Paisley, UK). Positive and negative controls were included in the extraction, and DNA was eluted with 80 µL of elution buffer. DNA was stored at + 4 °C prior to analysis. qPCR was performed in duplicate on an Applied Biosystems 7500 Fast instrument using the King et al. assay targeting ASFV VP72 [25], and genome copies per mL or mg were determined by comparison to an ASFV VP72 plasmid standard [25].

### Statistical analysis

All the statistical analyses and data visualisations were performed with GraphPad Prism Version 7.0 (GraphPad Software, La Jolla, CA, USA). The statistical tests applied to each dataset are indicated in the figure legends.

## Results

### Clinical findings

Wild boar and domestic pigs were euthanised at predetermined time points: 1, 2, 3, and 5 pi (Figure 1A). At 5 pi, four animals from each subspecies were retained until they reached humane clinical endpoints. All wild boar reached their humane endpoint on day 6 pi, whereas for domestic pigs, this endpoint was reached on day 9 pi (Figure 1B).

Throughout the study, both the infected domestic pig and wild boar groups exhibited a minimal to modest increase in mean weights compared to their pre-infection weights, but this increase was markedly lower than the weight gain of the uninfected animals (Figure 1C). Specifically, the weight of the infected domestic pigs increased less than 10% over a 9-day period, whereas that of the uninfected pigs increased from 20 to 40% by the end of the experiment. The weight of infected wild boar, which were on average 9 kg lighter than those of domestic pigs at the start of the study, remained relatively stable at approximately 15%, while the weight of uninfected boar increased by 20% to 30% by the end of the experiment. In summary, while no significant weight gain was observed among the infected domestic pigs and wild boar, the infected animals exhibited less weight gain than did the uninfected animals (the individual weights of the infected and uninfected animals are shown in Additional file 1).

**Figure 2:**
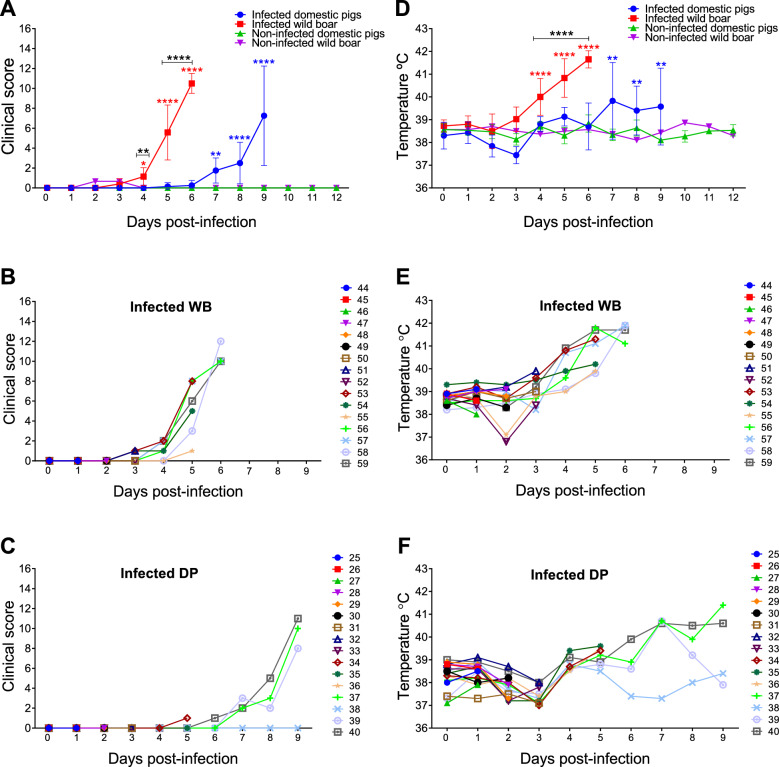
**Kinetics of clinical signs and rectal temperatures**. Statistically significant differences in clinical scores (**A**) and rectal temperatures (**D**) at different days after infection (mean ± SD) between the experimental groups were assessed by two-way ANOVA. Black asterisks indicate statistically significant differences between infected wild boar and infected domestic pigs. Statistically significant differences in rectal temperatures and clinical scores (mean ± SD) within each experimental group at different days after infection compared to pre-infection values were assessed by one-way ANOVA. Statistical differences are represented by red (infected boar group) and blue (infected domestic pig group) asterisks. Individual kinetics of clinical scores (**B**, **C**) and rectal temperatures (**E**, **F**) in infected wild boar (WBs) and domestic pigs (DPs). Day post-infection (x-axis); temperature and clinical score (y-axis); variables of significance (*P ≤ 0.05; **P ≤ 0.01; ***P ≤ 0.001; ****P ≤ 0.0001).

Compared with domestic pigs, wild boar developed clinical disease earlier, although their clinical scores were comparable at the humane endpoint (Figure 2A). Mild and nonspecific signs were observed starting on day 4 pi in 5 out of 7 infected wild boar (Figure 2B); however, no signs were detected in domestic pigs (Figure 2C) at this time point (*p* ≤ 0.01; Figure 2A). These signs included a reduction in liveliness, apathy, and leftover food after feeding. Subsequently, on day 5 pi, the clinical scores increased significantly, with signs such as lethargy, weakness, recumbency, difficulty or unwillingness to stand, lack of coordination or a stiff gait, loss of appetite, increased respiratory rate, ocular discharge, and soft faeces becoming evident. The four wild boar that were not euthanised at predefined time points reached the predetermined clinical endpoint on day 6 pi and were euthanised with high clinical scores.

In contrast, none of the domestic pigs euthanised up to day 5 pi exhibited notable clinical signs (Figure 2C). With the exception of one pig (#38), the remaining inoculated animals that survived until day 9 pi (#37, #39, and #40) exhibited a significant increase in clinical scores starting from day 7 pi, with clinical signs similar to those described for the infected wild boar. Starting at 8 pi, these animals also exhibited erythematous pinnae, kyphosis, and tremors, with the highest scores (a maximum of 11) recorded on 9 pi. Pig #38 did not reach the clinical endpoint scores but was euthanised to avoid single housing. The mock-inoculated domestic pigs and wild boar remained healthy and displayed no clinical signs throughout the study period (Additional file 2).

As part of clinical monitoring, rectal temperatures were recorded to assess disease development. Wild boar exhibited pyrexia earlier than domestic pigs after virus inoculation (Figure 2D). Among the infected wild boar, the mean rectal temperature began to rise on day 3 pi, with a significant increase compared to pre-inoculation values observed by day 4 pi (*p* ≤ 0.0001). The temperatures peaked on day 6 pi, averaging 41.7 °C (Figure 2D). Notably, starting at 4 pi, three of the seven remaining infected wild boar developed hyperthermia (>40.5 °C) (Figure 2E). The temperatures of all four wild boar that survived until day 6 (animals #56, #57, #58, and #59) exceeded 41.5 °C from day 5 pi, with peaks reaching 41.9 °C (Figure 2E).

In the case of the infected domestic pigs, the mean temperature significantly increased compared to the pre-inoculation value (*p* ≤ 0.01) from day 7 pi and remained elevated until day 9 pi, when the humane endpoint was reached (Figure 2D). The average temperatures ranged between 39.4 and 39.8 °C. Three of the four domestic pigs that survived until day 9 pi (animals #37, #39, and #40) exhibited hyperthermia (> 40.5 °C), with the highest temperature reaching 41.4 °C (Figure 2F). Pig #38 remained normothermic. The mock-infected animals maintained temperatures ranging between 38 and 39 °C, which is within the expected range (Additional file 2).

In summary, the incubation period was shorter in infected wild boar (4 days) than in infected domestic pigs (7 days). Infected wild boar exhibited higher temperatures in the early stages after infection, while domestic pigs only developed elevated temperatures starting from day 7 pi, with rectal temperatures notably higher in the wild boar. Clinical disease was observed in the infected wild boar from day 4 pi, with the most significant differences noted on day 6 pi. In contrast, domestic pigs developed clinical disease from day 7 pi, with the most significant differences observed on day 9 pi. The duration of clinical courses to the humane endpoint was similar between the groups of infected animals that were not euthanised at the established time points (3 days). However, while all four infected wild boar reached high clinical scores leading to the humane endpoint, only three out of the four infected domestic pigs reached the clinical endpoint.

### Macroscopic pathology

**Figure 3:**
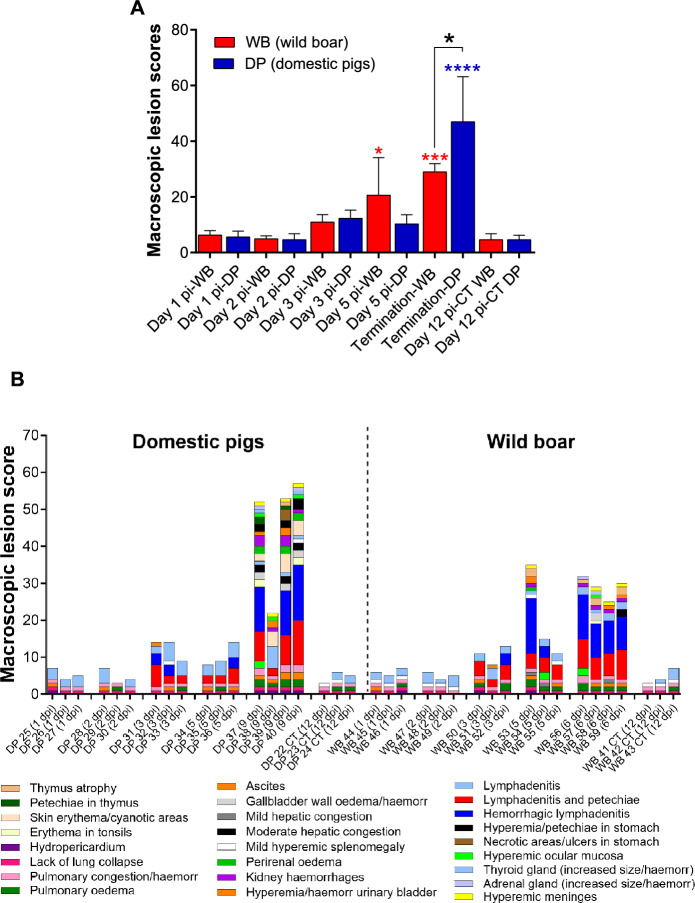
**Macroscopic evaluation of lesions. A** Mean ± SD of the cumulative macroscopic scores (y-axis) in each group of domestic pigs or wild boar euthanised on different days after infection. Uninfected animals (CT) euthanised on day 12 pi are also shown (x-axis). Statistical analysis was performed by one-way ANOVA. Black asterisks indicate statistically significant differences between the two groups of infected animals (wild boar and domestic pigs) euthanised on different dates, while red or blue asterisks indicate statistically significant differences with respect to the uninfected wild boar or domestic pig group; significant variables (*P ≤ 0.05; **P ≤ 0.01; ***P ≤ 0.001; ****P ≤ 0.0001);** B** Macroscopic scoring of lesions. Macroscopic lesions were scored and documented individually, representing the cumulative score observed in the different organs of the infected animals examined; macroscopic lesion score (y-axis); (x-axis): individual representation of animals evaluated in each experimental group and euthanised on different dates after infection. Uninfected animals (CT) euthanised on day 12 pi are also shown.

Starting at day 3 pi, both infected domestic pigs and infected wild boar exhibited a small increase in macroscopic scores compared to those of noninfected animals (Figure 3A). These scores were significantly different from those of the control animals on day 5 pi and at the end of the study (day 6 pi) in the infected wild boar group. In the infected domestic pig group, these differences were observed only at the end of the study (day 9 pi). However, the difference was not statistically significant; the macroscopic scores were slightly greater in the wild boar group than in the domestic pig group on day 5 pi. However, on the day of termination at the clinical endpoint, the pathology scores of domestic pigs were significantly greater than those of wild boar (*P* < 0.05).

**Figure 4:**
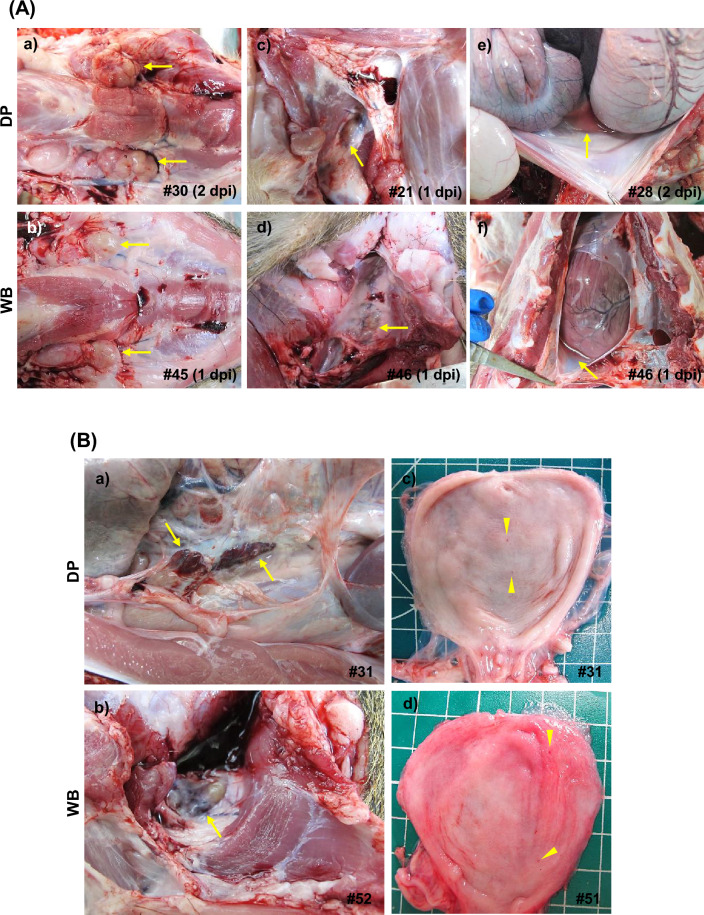
**Representative macroscopic lesions. A** 1 and 2 dpi. There was a mild increase in the size of the submandibular (a, b) and retropharyngeal (c, d) lymph nodes; mild ascites (e) and mild hydropericardium (f); **B** 3 dpi. Haemorrhagic sublumbar (a) and medial retropharyngeal (b) lymph nodes; petechial haemorrhages on the urothelium (c, d). DP: domestic pig; WB: wild boar.

The mock-inoculated domestic pigs and wild boar had only mild and nonspecific macroscopic changes that were attributed to the euthanasia procedures, including a lack of pulmonary collapse, lung congestion, alveolar oedema, splenomegaly, and occasional lymphadenopathy, mainly in the submandibular and retropharyngeal lymph nodes (Figure 3B). Both domestic pigs and wild boar euthanised on days 1 and 2 pi displayed nonspecific macroscopic findings similar to those of the control animals, although some animals (domestic pigs #25 and #28 and wild boar #44 and #46) also had mild hydropericardium and ascites (Figure 4A).

Along with the mild ascites and hydropericardium observed in some animals, all domestic pigs and wild boar euthanised on day 3 pi displayed mild lymphadenomegaly with petechiae in the submandibular, medial retropharyngeal, gastrohepatic, renal, and ileocecal lymph nodes. In addition, two domestic pigs (#31 and #32) and one wild boar (#52) also had haemorrhagic medial retropharyngeal and sub-lumbar lymph nodes. Domestic pig #31 and wild boar #51 also showed petechiation on the urothelium (Figure 4B).

**Figure 5:**
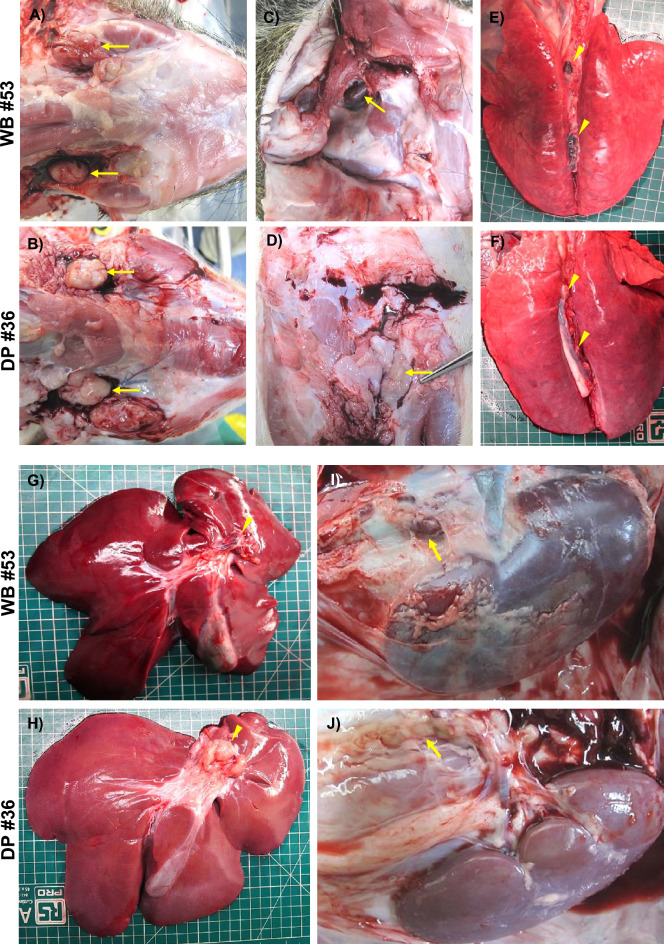
**Representative macroscopic images of lesions at 5 dpi.** Lesions observed in wild boar were more severe than those in domestic pigs. Note how the submandibular (**A**), retropharyngeal (**C**), mediastinal (**E**), gastrohepatic (**G**) and renal (**I**) lymph nodes of wild boar showed haemorrhagic lesions (from petechiae to severe diffuse haemorrhages) compared to those of domestic pigs, which showed only mild to moderate lymphadenopathy (**B**, **D**, **F**, **H**, **J**). Additionally, moderate hepatic congestion was observed in wild boar (**G**) compared to domestic pigs (**H**). DP: domestic pig; WB: wild boar.

In domestic pigs euthanised on day 5 pi, the macroscopic lesions were quite similar to those described in domestic pigs on day 3 pi. In contrast, the lesions were much more severe in the wild boar, especially in animal #53 (Figure 5) and, to a lesser extent, in animal #54. Wild boar #54 exhibited hepatic and splenic congestion and lymphadenomegaly with petechiae (only the gastrohepatic lymph node had severe haemorrhages). In contrast, wild boar #53 displayed a wider range of lesions, including hydropericardium, pulmonary congestion, alveolar oedema, ascites, thymic atrophy, hepatic congestion (Figure 5G), perirenal oedema, petechiae in the renal cortex and urinary bladder, meningeal hyperaemia, and severe generalised haemorrhagic lymph nodes (retropharyngeal, gastrohepatic, renal, mediastinal, and sub-lumbar; Figures 5A, C, E, G, I). The four wild boar that reached a humane endpoint on day 6 pi (#56, #57, #58, and #59) all displayed macroscopic findings and lesion scores similar to those described for wild boar #53, which was euthanised on day 5 pi. Wild boar #57 also showed hyperaemia of the ocular mucosa, erythematous palatine tonsils, and gallbladder wall edema with petechiae on the mucosa.

**Figure 6:**
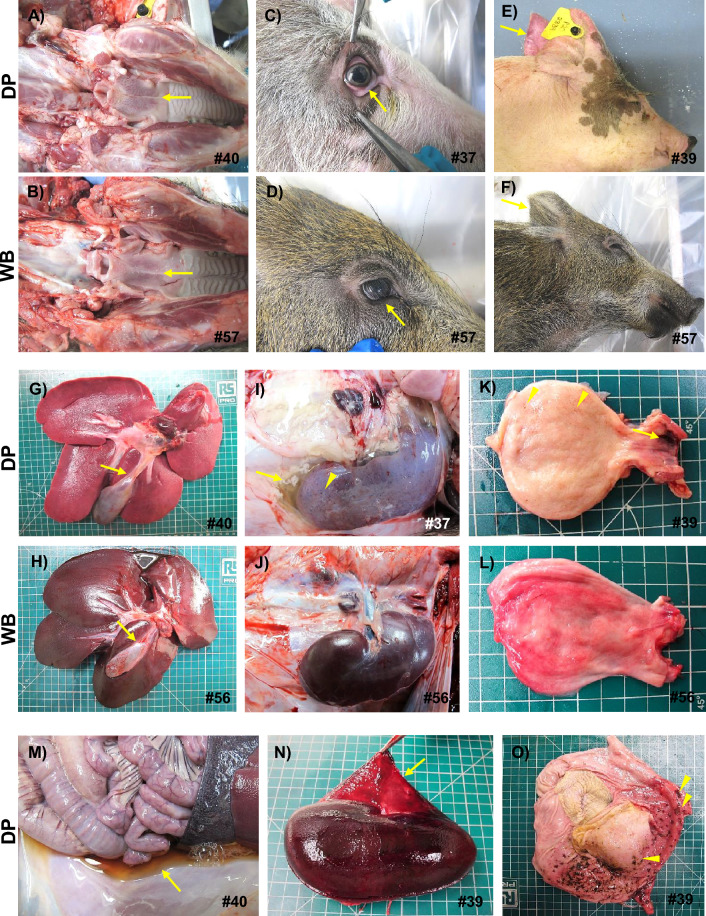
**Representative macroscopic lesions observed at the humane endpoint (6 dpi in wild boar and 9 dpi in domestic pigs).** Domestic pigs had a wider range of lesions and greater severity than did wild boar. Note the differences between domestic pigs and wild boar in terms of erythema on palatine tonsils (**A, B**), ocular mucosal hyperaemia (**C, D**) or cutaneous erythema on pinnae (**E, F**). Lesions such as hepatic congestion (G, H), gallbladder wall oedema (arrow; **G, H**), perirenal oedema (arrow; **I, J**), renal haemorrhages (arrowheads; **I, J**) and haemorrhages in the urinary bladder (**K, L**) were also more severe and more frequently detected in domestic pigs. Domestic pigs also had severe ascites with abundant amber fluid (**M**), severe renal interstitial haemorrhages involving the renal capsule (arrow; **N**) and congested gastric mucosa with petechiae and ecchymosis, as well as scattered necrotic areas (arrowheads; **O**).

Three of the four domestic pigs that reached the humane endpoint on day 9 pi (#37, #39, and #40) had a wider range of lesions, with higher scores than those observed in wild boar euthanised on day 6 pi, and in some instances lesions were only present in the domestic pigs but not in the wild boar (Figure 6). These included severe erythematous palatine tonsils (Figure 6A), hyperaemic ocular mucosa (Figure 6C), cutaneous erythema, and cyanosis in various areas, such as the pinnae (Figure 6E), snout, periorbital region, neck, ventral chest, dorsolateral skin areas, abdomen, scrotal sac, perianal area, tail, and distal limbs. These skin lesions were not observed in any of the wild boar on day 6 pi. Lesions such as hydropericardium, pulmonary congestion, alveolar oedema, hepatic congestion (Figure 6G), gallbladder wall oedema (arrow, Figure 6G), ascites (Figure 6M), perirenal oedema (arrow, Figure 6i), renal haemorrhages (arrowhead, Figure 6I; Figure 6N), hyperaemia, and petechiae in the urinary bladder (Figure 6K) or meningeal hyperaemia were also more severe and more frequently detected in domestic pigs. In addition, there was gastric mucosal congestion, petechiae and ecchymoses, as well as scattered necrotic areas in one pig (#39) (Figure 6O). For lymphoid organs such as the spleen, lymph nodes, and thymus, the severity of lesions was equivalent to that observed in wild boar, with mild to moderate splenomegaly with congestion and thymic atrophy in some animals, as well as generalised, severe haemorrhagic lymphadenitis. The fourth domestic pig, #38, displayed only mild lesions, such as cutaneous erythema and cyanosis, hyperaemic ocular mucosa, hyperaemia, and petechiae in the urinary bladder; ascites; meningeal hyperaemia; and mild lymphadenomegaly with oedema but without severe haemorrhagic changes.

### Viral load in blood samples and nasal and rectal swabs

**Figure 7:**
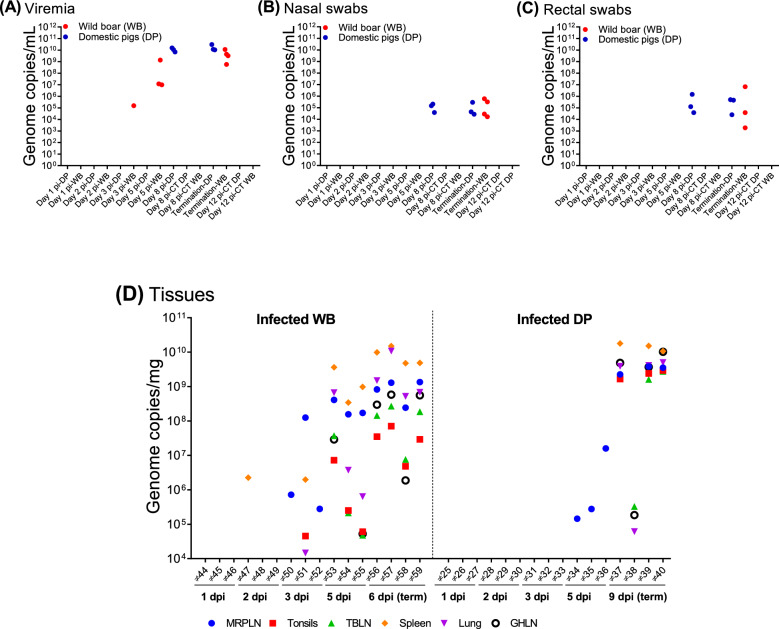
**Viral genome copies in blood samples (A), nasal swabs (B) and rectal swabs (C) taken from infected domestic pigs and wild boar**. The individual values obtained are shown. Viral genome copies were determined by qPCR and are represented as the total number of genome copies per millilitre (y-axis); (x-axis): group of domestic pigs or wild boar euthanised or sampled on different dates after infection. Uninfected animals (CT) euthanised on day 12 pi are also shown; **(D)** virus genome copies in tissue samples taken from infected domestic pigs and wild boar; (y-axis): genome copies per milligram; (x-axis): individual identification of each domestic pig (DP) or wild boar (WB) euthanised on different dates after infection; (term): termination date after reaching the humane endpoint; MRPLN: medial retropharyngeal lymph node; TBLN: tracheobronchial lymph node; GHLN: gastrohepatic lymph node.

Blood samples from both domestic pigs and wild boar tested negative for ASFV by qPCR before virus inoculation and on days 1 and 2 pi (Figure 7A). In wild boar, a significant amount of the virus genome was detected in blood samples from one animal (*n* = 1/3) euthanised on day 3 pi (#51; 1.53 × 10^5^ copies/mL) and from all three animals (*n* = 3/3) euthanised on day 5 pi (up to 1.38 × 10^9^ copies/mL, #53). These two animals (#51 and #53) also exhibited the highest clinical scores and temperatures among all animals euthanised on days 3 and 5 pi (Figures 2B and E). Wild boar that reached a humane endpoint on day 6 pi (#56, #57, #58, and #59) tested positive for the virus, showing higher viral loads (up to 1.13 × 10^10^ copies/mL). In contrast, no virus genome was detected in any of the blood samples from the domestic pigs euthanised on day 3 or 5 pi. Among the domestic pigs that reached a humane endpoint on day 9 pi, only three (#37, #39, and #40) were viraemic from day 8 pi. The levels of viremia in the domestic pigs were high (up to 1.56 × 10^10^ copies/mL in #40 on day 9 pi) and similar to those detected in wild boar at the humane endpoint.

The level of virus shedding was evaluated by nasal and rectal swabs. No viral genome was detected in any of the swabs taken from virus-inoculated wild boar or domestic pigs euthanised up to day 5 pi (Figures 7B and C). However, high viral loads were detected in nasal swabs taken from the four wild boar euthanised on day 6 pi (up to 5.93 × 10^5^ copies/mL) and in 3 of the 4 domestic pigs euthanised at 9 dpi (up to 2.94 × 10^5^ copies/mL), except for the domestic pig #38, which tested negative (Figure 7B). In the rectal swabs, the virus genome was detected in only 3 of the 4 wild boar and domestic pigs euthanised on days 6 and 9 pi, respectively (Figure 7C). An exception was wild boar #58, which also had the lowest virus loads in blood and nasal swabs. Both swabs of domestic pig #38 tested negative. At the time of termination, virus genome loads in nasal and rectal swabs were comparable between wild boar and domestic pigs that tested positive, ranging between 10^5^ and 10^6^ copies/mL. Blood samples and swabs (nasal and rectal) collected on 8 and 12 pi from mock-inoculated control animals tested negative for the virus genome.

### Viral load in tissue samples

Viral loads in the palatine tonsils, right cranial lobe of the lungs, spleen, medial retropharyngeal, tracheobronchial, and gastrohepatic lymph nodes were assessed using qPCR. The virus genome was detected earlier in the wild boar than in domestic pigs, with detection as early as day 2 pi in one wild boar, while domestic pigs only tested virus-positive by day 5 pi (Figure 7D).

In the wild boar, the viral genome was detected in the spleen of one animal (#47) on day 2 pi, with a viral load of 10^6^ copies/mg. At 3 dpi, all three wild boar were positive for the virus genome in at least one tissue, with consistent detection of the virus in the medial retropharyngeal lymph node (MRPLN) in all three animals. Animal #51 had the highest viral load, approximately 10^8^ copies/mg. Similarly, on day 5 pi, the virus genome was detected in all tested tissues from wild boar, particularly with high viral loads in the spleen (10^8^ to 10^9^ copies/mg) of MRPLN (10^9^ to 10^10^ copies/mg). In contrast, in domestic pigs, the virus was first detected at 5 pi and was detected only in the MRPLN (10^5^ to 10^7^ copies/mg) at that time.

In both wild boar and domestic pigs euthanised on day 6 and 9 pi, respectively, the viral genome was detected in all tested tissues. The MRPLN, spleen, and lung had the highest viral loads (10^8^ to 10^10^ copies/mg). However, in wild boar, the viral loads detected in the palatine tonsils (10^5^ to 10^7^ copies/mg), gastrohepatic lymph nodes (10^6^ to 10^9^ copies/mg), and tracheobronchial lymph nodes (10^5^ to 10^8^ copies/mg) were generally 10^1^ to 10^3^ times lower than those detected in domestic pigs, with the most noteworthy difference observed in the tonsils.

## Discussion

The present in vivo experimental findings demonstrated that wild boar were considerably more susceptible to infection than domestic pigs following intranasal exposure to the highly virulent isolate Armenia 2007, as characterised by an earlier onset of viremia and detection of the virus in tissues, a shorter incubation period and higher body temperatures and clinical scores. This finding concurs with previous studies that pointed to a greater susceptibility of wild boar after oronasal infection [7–9]. Only one study to date, with a similar experimental approach, has studied the kinetics of disease in both subspecies after oronasal infection. However, although that study used a moderately virulent Baltic isolate, it also revealed a greater susceptibility of wild boar to ASFV [17].

In line with our study, previous research has consistently shown that wild boar develop clinical signs shortly after oronasal infection. Highly virulent strains from the Caucasus region, typically within 2 to 5 days post-infection [7, 8] and between 3 and 4 days post-infection following oronasal infection of wild boar with highly virulent isolates such as Belgium 2018/1 [18], or moderately virulent Baltic isolates such as Estonia 2014 [9, 17]. Unexpectedly, the results also revealed a lower weight gain in infected wild boar than in uninfected wild boar.

The period between the onset of clinical signs and the humane endpoint remained consistent at 3 days for both groups of infected animals that had to be euthanised on day 6 and 9 pi. While all four infected wild boar necessitated euthanasia due to high clinical scores, only three out of four infected domestic pigs reached this predefined endpoint. Previous research [18] showed that oronasal infection of 1- to 2-year-old wild boar with the highly virulent Belgium 2018/1 isolate resulted in deaths between 8 and 10 days post-infection, leading to slightly longer clinical courses, possibly due to increased resistance in older animals [26]. Conversely, and in line with our results, younger wild boar at 9 weeks of age that were oronasally infected with highly virulent strains from the Caucasus region survived for 5 to 9 days [7, 10]. For domestic pigs, experiments with weanlings (8 to 10 weeks old) infected intranasally with the highly virulent Georgia 2007 isolate [27] or oronasally with the highly virulent Belgium 2018/1 isolate [19] showed incubation periods of 4 to 5 days, and survival times (8 to 10 days post-infection) were consistent with our study. However, variations were observed when, in the same experiment [19], 50% of 18-week-old domestic pigs inoculated intranasally with Belgium 2018/1 either died between day 7 and 14 pi or recovered. On the other hand, limited research has indicated that the age of wild boar does not affect the early onset of clinical signs following infection [28].

For domestic pigs, experiments with intranasally infected weanlings (8 to 10 weeks old) with the highly virulent Georgia 2007 isolate [27] or oronasally infected with the highly virulent Belgium 2018/1 isolate [19] showed incubation periods of 4 to 5 days, and survival times (8 to 10 days post-infection) were consistent with our study.

The available literature describing macroscopic changes observed in experimental infections with isolates belonging to genotype II of wild boar and/or domestic pigs usually describes findings in advanced stages of the disease in animals, including at death or at the humane endpoint. This provides limited information that neither provides information on disease progression nor reflects the spectrum of disease severity, which can be quite diverse in field infections [7–10, 16–19, 27]. To address this gap in knowledge about host‒virus interactions and disease dynamics in the critical period immediately after infection, our experimental approach involved sequential euthanasia of animals beginning on day 1 pi. During this process, we systematically assessed the animals using established macroscopic evaluation protocols [24, 29] to provide more precise information on affected organs, lesion types and severity to establish differences between animals and experimental groups in the early stages after infection. Such protocols could also be useful and easy-to-use tools for the early detection of disease during outbreaks, requiring only minimal training from veterinarians.

Notable macroscopic lesions were detected as early as day 3 pi, and both infected domestic pigs and wild boar showed characteristic lesions associated with acute ASF [24, 29]. These included lymphadenomegaly with petechiae in the submandibular, medial retropharyngeal, gastrohepatic, renal, and ileocecal lymph nodes. The medial retropharyngeal and sublumbar lymph nodes exhibited diffuse haemorrhages in some patients. Previous studies have primarily focused on the gastrohepatic and renal lymph nodes, where haemorrhagic lesions were observed in wild boar at different stages, ranging from as early as day 4 pi in experiments with scheduled sacrifices [17] to days 6–7 pi, when animals reached a humane endpoint [8, 16, 18]. Other lymph nodes, such as the medial retropharyngeal lymph node (MRPLN), had not been assessed in prior studies. In our investigation, the MRPLNs consistently exhibited high viral loads along with macroscopic lesions. Given that lymphoid tissues such as tonsils and the MRPLN are responsible for immune surveillance of the oronasal environment, the gross lesions detected imply an early role in virus‒cell interactions, the host immune response and virus transmission.

There were significant differences in macroscopic lesions on day 5 pi. Wild boar displayed more severe and extensive changes, while in domestic pigs, lesions were milder. In contrast, domestic pigs that reached the humane endpoint exhibited markedly greater macroscopic scores than did wild boar euthanised at the same clinical endpoint, with a greater number of affected organs and more severe and extensive haemorrhagic lesions. Accordingly, while wild boar develop severe disease more rapidly and reach the clinical endpoint earlier, they develop less severe macroscopic lesions, underscoring their greater susceptibility to ASFV.

The greater susceptibility of wild boar to early virus replication and spread could be related to their virological profile. While none of the infected domestic pigs euthanised up to day 5 pi showed viremia, one wild boar (#51) exhibited viremia on day 3 pi. In previous studies, oronasal infections with high-dose highly virulent strains originating from the Caucasus region [7, 10] or with moderately virulent isolates originating from the Baltic region [9, 16, 17] resulted in viremia at 4 to 5 days pi in wild boar. In oronasal infections of domestic pigs with the moderately virulent Baltic isolates viremia developed by 3 to 4 days pi [9, 16, 17], whereas with the highly virulent isolates Belgium 2018/1 or Georgia 2007 viremia developed by 6 to 7 days pi [19, 27], the latter more in line with our results. In our study, both wild boar and domestic pigs consistently showed the presence of the virus genome in the MRPLN on 3 and 5 pi, respectively, which underlines the role of the MRPLN in early virus replication following intranasal infection before viremia and demonstrates differences in virus replication kinetics between the two subspecies, with a delay in domestic pigs. However, notably, the tonsils did not serve as a main organ for viral genome detection on day 3 in wild boar or day 5 in domestic pigs, despite this organ being conventionally recognised as one of the initial key sites for ASFV replication and diagnostic sampling [30, 31].

As mentioned, the haemorrhagic MRPLN exhibited high viral loads at early stages after infection, demonstrating its importance, together with tonsils, for the early detection of ASFV during post-mortem examinations. Conversely, in lymph nodes with less severe haemorrhages, such as tracheobronchial or gastrohepatic lymph nodes, the early presence of the viral genome was not detected. Earlier studies on domestic pigs infected with highly virulent genotype I isolates suggested that the presence of haemorrhagic lesions in lymph nodes at 3 dpi was not a result of viral replication in endothelial cells but rather the consequent activation and disruption of endothelial cells mediated by cytokines [21, 32]. A similar pathogenic mechanism may explain the presence of haemorrhagic lesions in our animals before the detection of viral antigens in the lymph nodes, although further studies are required to confirm this hypothesis.

The viral genome remained undetected in nasal and rectal swabs from euthanised wild boar and domestic pigs up to day 5 pi. However, in the wild boar, moderate levels of the viral genome were detected on the day of termination (day 6 pi) and on days 8 and 9 pi in domestic pigs. This indicates limited virus shedding until shortly before death, coinciding with the peak viremia. These results are consistent with previous reports where low viral genome loads were detected in oral and faecal swabs from infected wild boar [7, 10, 16] and domestic pigs [9] between 3 and 5 pi, with higher genome loads detected in most infected animals from 6 pi onwards. Given that only a low viral dose is required to infect an animal [8], low to moderate virus shedding, as detected in our study, may suggest the potential for disease transmission through nasal secretions and faecal excretions, although only efficiently in the later stages of the disease. This means that in earlier stages of infection, transmission probably occurs more efficiently through contact with contaminated blood or tissues, biological materials where the virus is able to maintain its infectivity for several months, especially at low temperatures [33]. In this regard, wild boar carcasses persisting in the environment have been identified as a source of infection where the virus can persist for several months, and together with cannibalism in wild boar, this is considered to play a substantial role in the ASF epidemic. [34].

In conclusion, our results demonstrate that wild boar are more susceptible to ASFV than are domestic pigs. The wild boar exhibited shorter incubation periods, earlier onset of clinical signs and hyperthermia, and more rapid development of severe and extensive haemorrhagic lesions. Moreover, wild boar displayed earlier viremia and an earlier presence of the virus genome in target organs. Notably, lymphoid tissues within the oronasal tract, including the medial retropharyngeal lymph nodes, were identified as key portals for ASFV infection and the establishment of systemic infection and disease following intranasal exposure. Recognising these different host responses in wild boar and domestic pigs is critical for designing effective control strategies such as vaccine development and understanding the dissemination of ASFV in different host populations. Further research is needed to uncover the genetic and immunological factors contributing to these differences.

### Supplementary Information

**Additional file 1. Individual weights of infected (A, B) and noninfected (C, D) domestic pigs (DP) and wild boar (WB).** Day post-infection (x-axis); Weight (y-axis).

**Additional file 2. Individual kinetics of clinical scores (A, B) and rectal temperatures (C, D) in uninfected domestic pigs (DP) and uninfected wild boar (WB) used as controls.** Day post-infection (x-axis); temperature and clinical score (y-axis).

## Data Availability

The datasets used and/or analysed during the current study are available from the corresponding author upon reasonable request. The dataset supporting the conclusions of this article is included within the article (and its supplementary information files).

## References

[1] World Organization for Animal Health (WOAH) (2024) African swine fever (ASF)—situation report 46. https://www.woah.org/app/uploads/2024/02/asf-report46.pdf. Accessed 29 Jan 2024

[2] Alonso C, Borca M, Dixon L, Revilla Y, Rodriguez F, Escribano JM (2018). ICTV virus taxonomy profile: Asfarviridae. J Gen Virol.

[3] Quembo CJ, Jori F, Vosloo W, Heath L (2018). Genetic characterization of African swine fever virus isolates from soft ticks at the wildlife/domestic interface in Mozambique and identification of a novel genotype. Transbound Emerg Dis.

[4] Sánchez-Vizcaíno JM, Mur L, Gomez-Villamandos JC, Carrasco L (2015). An update on the epidemiology and pathology of African swine fever. J Comp Pathol.

[5] Olesen AS, Lohse L, Boklund A, Halasa T, Gallardo C, Pejsak Z, Belsham GJ, Rasmussen TB, Bøtner A (2017). Transmission of African swine fever virus from infected pigs by direct contact and aerosol routes. Vet Microbiol.

[6] Tran XH, Le TTP, Nguyen QH, Do TT, Nguyen VD, Gay CG, Borca MV, Gladue DP (2022). African swine fever virus vaccine candidate ASFV-G-ΔI177L efficiently protects European and native pig breeds against circulating Vietnamese field strain. Transbound Emerg Dis.

[7] Gabriel C, Blome S, Malogolovkin A, Parilov S, Kolbasov D, Teifke JP, Beer M (2011). Characterization of African swine fever virus Caucasus isolate in European wild boar. Emerg Infect Dis.

[8] Pietschmann J, Guinat C, Beer M, Pronin V, Tuscher K, Petrov A, Keil G, Blome S (2015). Course and transmission characteristics of oral low-dose infection of domestic pigs and European wild boar with a Caucasian African swine fever virus isolate. Arch Virol.

[9] Zani L, Forth JH, Forth L, Nurmoja I, Leidenberger S, Henke J, Carlson J, Breidenstein C, Viltrop A, Höper D, Sauter-Louis C, Beer M, Blome S (2018). Deletion at the 5'-end of Estonian ASFV strains associated with an attenuated phenotype. Sci Rep.

[10] Blome S, Gabriel C, Dietze K, Breithaupt A, Beer M (2012). High virulence of African swine fever virus caucasus isolate in European wild boar of all ages. Emerg Infect Dis.

[11] Sehl-Ewert J, Deutschmann P, Breithaupt A, Blome S (2022). Pathology of African swine fever in wild boar carcasses naturally infected with German virus variants. Pathogens.

[12] Guinat C, Reis AL, Netherton CL, Goatley L, Pfeiffer DU, Dixon L (2014). Dynamics of African swine fever virus shedding and excretion in domestic pigs infected by intramuscular inoculation and contact transmission. Vet Res.

[13] Vlasova N, Varentsova A, Shevchenko I, Zhukov I, Remyga SG, Gavrilova VL, Puzankova OS, Shevtsov AA, Zinyakov G, Gruzdev KN (2014). Comparative analysis of clinical and biological characteristics of African swine fever virus isolates from 2013 year Russian Federation. Br Microbiol Res J.

[14] Gallardo C, Nurmoja I, Soler A, Delicado V, Simón A, Martin E, Pérez C, Nieto R, Arias M (2018). Evolution in Europe of African swine fever genotype II viruses from highly to moderately virulent. Vet Microbiol.

[15] Gallardo C, Soler A, Nieto R, Cano C, Pelayo V, Sánchez MA, Pridotkas G, Fernandez-Pinero J, Briones V, Arias M (2017). Experimental infection of domestic pigs with African swine fever virus Lithuania 2014 genotype II field isolate. Transbound Emerg Dis.

[16] Nurmoja I, Petrov A, Breidenstein C, Zani L, Forth JH, Beer M, Kristian M, Viltrop A, Blome S (2017). Biological characterization of African swine fever virus genotype II strains from north-eastern Estonia in European wild boar. Transbound Emerg Dis.

[17] Sehl J, Pikalo J, Schäfer A, Franzke K, Pannhorst K, Elnagar A, Blohm U, Blome S, Breithaupt A (2020). Comparative pathology of domestic pigs and wild boar infected with the moderately virulent African swine fever virus strain "Estonia 2014". Pathogens.

[18] Pikalo J, Schoder ME, Sehl J, Breithaupt A, Tignon M, Cay AB, Gager AM, Fischer M, Beer M, Blome S (2020). The African swine fever virus isolate Belgium 2018/1 shows high virulence in European wild boar. Transbound Emerg Dis.

[19] Pikalo J, Schoder ME, Sehl-Ewert J, Breithaupt A, Cay AB, Lhoëst C, van Campe W, Mostin L, Deutschmann P, Roszyk H, Beer M, Blome S, Tignon M (2021). Towards efficient early warning: pathobiology of African swine fever virus "Belgium 2018/1" in domestic pigs of different age classes. Animals (Basel).

[20] Rodríguez-Bertos A, Cadenas-Fernández E, Rebollada-Merino A, Porras-González N, Mayoral-Alegre FJ, Barreno L, Kosowska A, Tomé-Sánchez I, Barasona JA, Sánchez-Vizcaíno JM (2020). Clinical course and gross pathological findings in wild boar infected with a highly virulent strain of African swine fever virus genotype II. Pathogens.

[21] Gomez-Villamandos JC, Bautista MJ, Sanchez-Cordon PJ, Carrasco L (2013). Pathology of African swine fever: the role of monocyte-macrophage. Virus Res.

[22] Howey EB, O’Donnell V, de Carvalho Ferreira HC, Borca MV, Arzt J (2013). Pathogenesis of highly virulent African swine fever virus in domestic pigs exposed via intraoropharyngeal, intranasopharyngeal, and intramuscular inoculation, and by direct contact with infected pigs. Virus Res.

[23] McCleary S, Strong R, McCarthy RR, Edwards JC, Howes EL, Stevens LM, Sánchez-Cordón PJ, Núñez A, Watson S, Mileham AJ, Lillico SG, Tait-Burkard C, Proudfoot C, Ballantyne M, Whitelaw CBA, Steinbach F, Crooke HR (2020). Substitution of warthog NF-κB motifs into RELA of domestic pigs is not sufficient to confer resilience to African swine fever virus. Sci Rep.

[24] Sánchez-Cordón PJ, Floyd T, Hicks D, Crooke HR, McCleary S, McCarthy RR, Strong R, Dixon LK, Neimanis A, Wikström-Lassa E, Gavier-Widén D, Núñez A (2021). Evaluation of lesions and viral antigen distribution in domestic pigs inoculated intranasally with African swine fever virus Ken05/Tk1 (genotype X). Pathogens.

[25] King DP, Reid SM, Hutchings GH, Grierson SS, Wilkinson PJ, Dixon LK, Bastos AD, Drew TW (2003). Development of a TaqMan PCR assay with internal amplification control for the detection of African swine fever virus. J Virol Methods.

[26] Post J, Weesendorp E, Montoya M, Loeffen WL (2022). Influence of age and dose of african swine fever virus infections on clinical outcome and blood parameters in pigs. Viral Immunol.

[27] Lohse L, Nielsen J, Uttenthal Å, Olesen AS, Strandbygaard B, Rasmussen TB, Belsham GJ, Bøtner A (2022). Experimental infections of pigs with African swine fever virus (genotype II); studies in young animals and pregnant sows. Viruses.

[28] Schulz K, Conraths FJ, Blome S, Staubach C, Sauter-Louis C (2019). African swine fever: fast and furious or slow and steady?. Viruses.

[29] Sánchez-Cordón PJ, Lean F, Bernard M, Núñez A, Netherton CL (2022). Necropsy procedures and evaluation of macroscopic lesions of pigs infected with African swine fever virus. African swine fever virus: methods and protocols, Chapter 2.

[30] Plowright W, Parker J, Staple RF (1968). The growth of a virulent strain of African swine fever virus in domestic pigs. J Hyg (Lond).

[31] Oura CA, Powell PP, Parkhouse RM (1998). African swine fever: a disease characterized by apoptosis. J Gen Virol.

[32] Carrasco L, Chacón-M de Lara F, de Martín los Mulas J, Gómez-Villamandos JC, Sierra MA, Villeda CJ, Wilkinson PJ (1997). Ultrastructural changes related to the lymph node haemorrhages in acute African swine fever. Res Vet Sci.

[33] Fischer M, Hühr J, Blome S, Conraths FJ, Probst C (2020). Stability of African swine fever virus in carcasses of domestic pigs and wild boar experimentally infected with the ASFV "Estonia 2014" isolate. Viruses.

[34] Cukor J, Linda R, Václavek P, Mahlerová K, Šatrán P, Havránek F (2020). Confirmed cannibalism in wild boar and its possible role in African swine fever transmission. Transbound Emerg Dis.

