# Supplementary figures and images for: Comparative evaluation of disease dynamics in wild boar and domestic pigs experimentally inoculated intranasally with the European highly virulent African swine fever virus genotype II strain “Armenia 2007”
Source: Vet Res. 2024 Jul 15;55:89. doi: 10.1186/s13567-024-01343-5 (PMC11247888; doi:10.1186/s13567-024-01343-5)

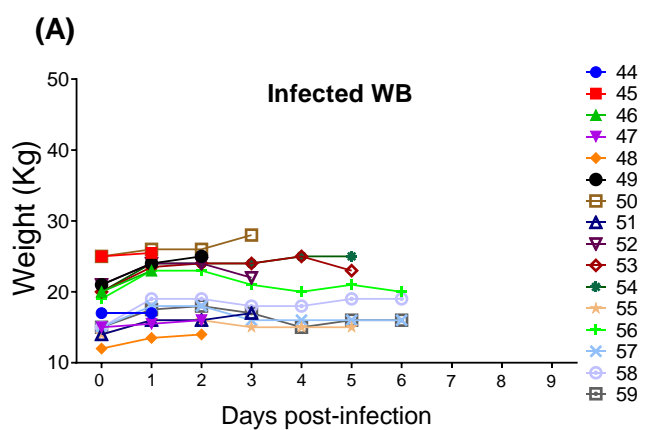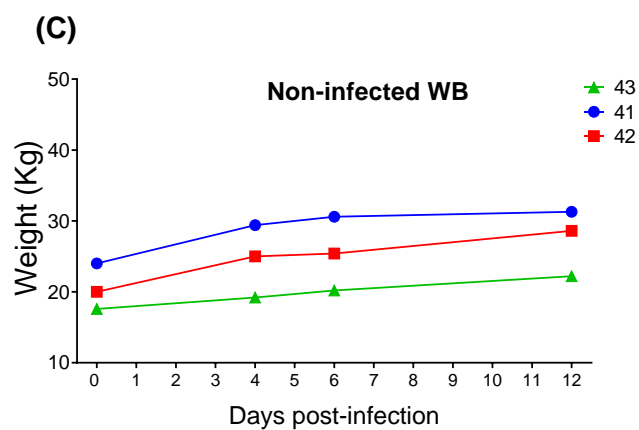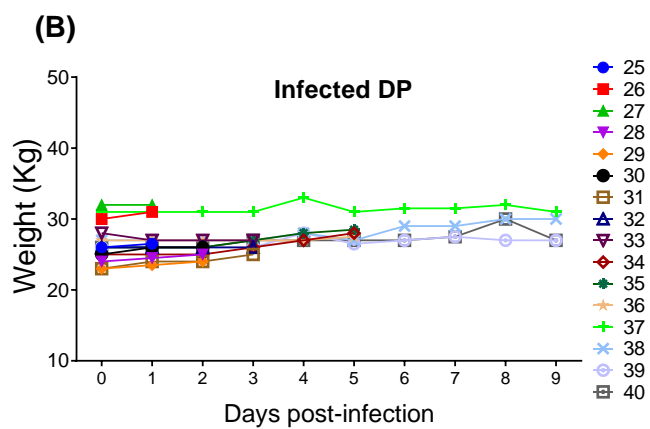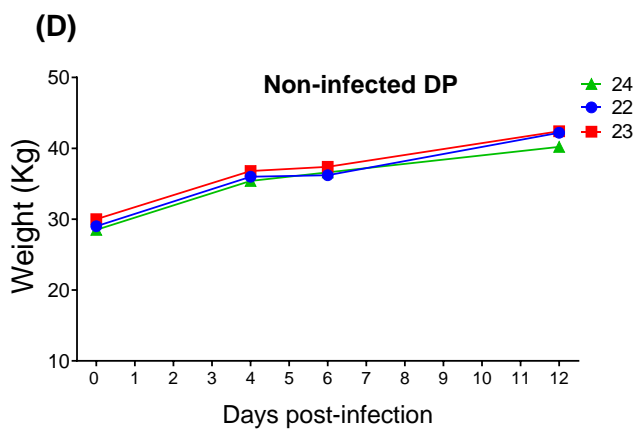

Supplement: Supplementary file 1 — Additional file 1. Individual weights of infected (A, B) and noninfected (C, D) domestic pigs (DP) and wild boar (WB). Day post-infection (x-axis); Weight (y-axis). [file 13567_2024_1343_MOESM1_ESM.pdf]

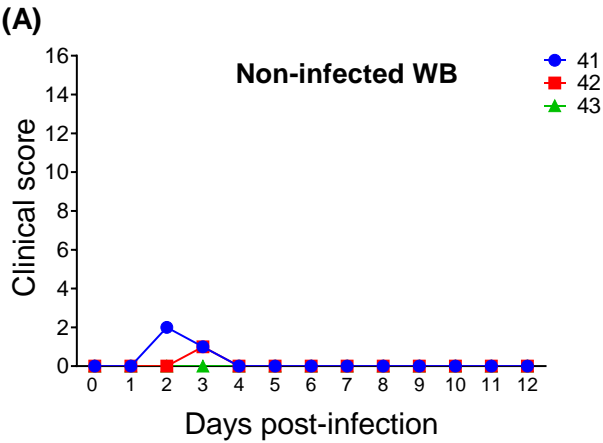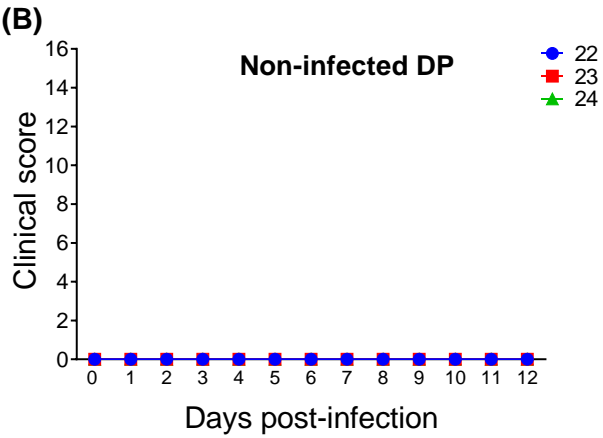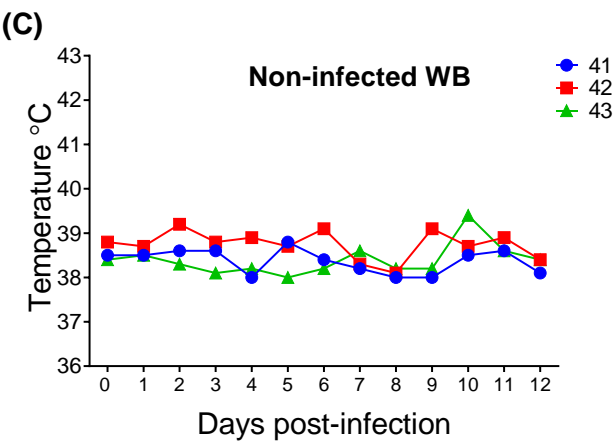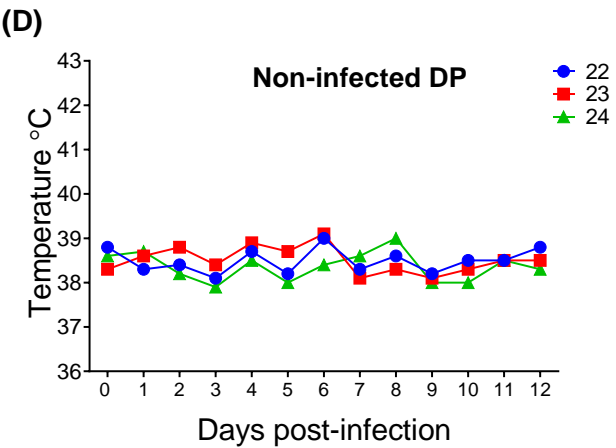

Supplement: Supplementary file 2 — Additional file 2. Individual kinetics of clinical scores (A, B) and rectal temperatures (C, D) in uninfected domestic pigs (DP) and uninfected wild boar (WB) used as controls. Day post-infection (x-axis); temperature and clinical score (y-axis). [file 13567_2024_1343_MOESM2_ESM.pdf]
